# Efficacy of ustekinumab, vedolizumab, or a second anti-TNF agent after the failure of a first anti-TNF agent in patients with Crohn’s disease: a multicentre retrospective study

**DOI:** 10.1186/s12876-022-02583-5

**Published:** 2022-12-01

**Authors:** Rayer Cassandra, Maria Nachury, Bourreille Arnaud, Roblin Xavier, Peyrin-Biroulet Laurent, Viennot Stephanie, Flamant Mathurin, Laharie David, Caron Bénédicte, Dewitte Marie, Siproudhis Laurent, Fumery Mathurin, Bouguen Guillaume

**Affiliations:** 1 CHU Rennes, University Rennes, 35000 Rennes, France; 2 CHU Lille, University of Lille, Lille, France; 3 CHU Nantes, Nantes, France; 4 CHU Saint-Etienne, Saint- Étienne, France; 5 Inserm U954 Deparment of Hepato-Gastroenterology, Department of Gastroenterology, Nancy University Hospital, Vandœuvre-Lès-Nancy, France; 6 CHU Caen, Caen, France; 7 Clinique Jules Vernes, Nantes, France; 8 CHU de Bordeaux, Hôpital Haut-Lévêque, Service d’Hépato-Gastroentérologie Et Oncologie Digestive, Université de Bordeaux, 33000 Bordeaux, France; 9 CHU Rennes, University Rennes, INSERM, CIC1414, Institute NUMECAN (Nutrition Metabolism and Cancer), 35000 Rennes, France; 10 Service d’Hépato-Gastroentérologie Et Oncologie Digestive, CHU Amiens Et PeriTox, UMR I0-I, Université de Picardie, Amiens, France; 11 Service Des Maladies de L’Appareil Digestif, 2 Rue Henri Le Guillou, 35033 Rennes Cedex, France

**Keywords:** Crohn’s disease, Anti-TNF, Vedolizumab, Ustekinumab, Treatment strategy, Second line

## Abstract

**Background:**

No study has performed a face-to-face comparison of biologics after the failure of the first anti-TNF agent in patients with Crohn’s disease (CD). The aim of the study was to compare the efficacy of biologics in this setting.

**Methods:**

Patients with CD who were refractory to a first anti-TNF agent, and treated with ustekinumab (UST), vedolizumab (VDZ), or a second anti-TNF drug as a second-line biological agent at 10 French tertiary centres from 2013 to 2019 were retrospectively included in this study.

**Results:**

Among the 203 patients included, 90 (44%) received UST, 42 (21%) received VDZ and 71 (35%) received a second anti-TNF agent. The first anti-TNF agent was discontinued due to a primary nonresponse in 42 (21%) patients. At weeks 14–24, the rates of steroid-free remission were similar between the UST, VDZ and second anti-TNF groups (29%, 38% and 44%, respectively, *p* = 0.15). With a mean follow-up of 118 weeks, drug survival was shorter for patients who received ustekinumab treatment (*p* = 0.001). In the case of trough level less than 5 µg/ml, patients treated with a second anti-TNF agent had a higher postinduction remission rate (*p* = 0.002), and drug survival (*p* = 0.0005). No other relevant factors were associated with treatment efficacy, including trough levels greater than 5 µg/ml.

**Conclusions:**

VDZ, UST and a second anti-TNF agent exhibit similar efficacy in the short term, as second-biological line treatment in patients with CD who are refractory to a first anti-TNF agent, but shorter drug maintenance is observed for patients treated with UST.

**Supplementary Information:**

The online version contains supplementary material available at 10.1186/s12876-022-02583-5.

## Introduction

Crohn’s disease (CD) is a chronic, and relapsing immune-mediated inflammatory bowel disease. Unremitting inflammation of the gut may lead to bowel damage and changes in disease behaviour, such as strictures, or fistulas that ultimately drastically alter the quality of life of patients, and increase disability [1, 2]. Therefore, the abrogation of mucosal inflammation should be the main objective of treatments, prompting international guidelines to recommend mucosal healing as the main target for treatment strategies, and physician decision-making [3].

Several biological treatments are currently available to treat CD, such as antitumour necrosis factor (TNF) agents (infliximab and adalimumab), or new biological therapies, including ustekinumab (anti-IL12/23 p40 antibody) and vedolizumab (anti-α4β7 integrin monoclonal antibody). All these treatments showed effectiveness as induction, and maintenance therapies when administered as first- or second-line biological therapies to patients with CD [4–9].No study has evaluated the placement of each of these treatments in the therapeutic strategy.

In France, the choice of the first-line biological therapy is guided by the sole reimbursement of anti-TNF agents for patients with moderate to severe CD. No reimbursement restriction is currently ongoing for a second- or third-line biologic, and several therapeutic choices are available following the failure of a first anti-TNF agent that includes a switch to a second anti-TNF agent, or a switch of the class for ustekinumab or vedolizumab. To date, a head-to-head trial comparing these different treatments when strictly used as a second-line biologic has not been conducted.

Recent multicentre retrospective studies observed conflicting results regarding the rate of clinical remission in patients treated with ustekinumab as compared to patients treated with vedolizumab [10–14]. Nevertheless, in these studies, ustekinumab, and vedolizumab were not strictly used as second-line therapies, since approximately 70% of patients had previously received at least two anti-TNF agents, making direct comparison difficult. Obviously, a comparison between a second anti-TNF agent and another therapeutic class is not available.

Similarly, no recommendation is currently available for the second-line biological therapy for patients with CD who are refractory to anti-TNF agents, and the choice is left to the clinician, and is based on a set of arguments considering their experience, the characteristics of the patient and his/her disease, and the reason for the failure of the first anti-TNF agent.

The aim of this study was therefore to compare the efficacy of ustekinumab, vedolizumab, and a second anti-TNF agent in patients with CD in whom a first anti-TNF agent failed, and to identify selection criteria for a second-line therapy that will assist physicians with decision-making.

## Materials and methods

### Patients and study design

All hospital records of adult patients (≥ 18 years) with (1) an established diagnosis of CD according to the usual criteria [15], (2) who experienced a failure of only one anti-TNF agent (adalimumab or infliximab), (3) were treated with ustekinumab or vedolizumab within 6 months of anti-TNF discontinuation, and (4) presented with an active disease at the time of ustekinumab, or vedolizumab introduction were reviewed at 10 referral GETAID centres in France (Nantes, Lille, Amiens, Saint-Etienne, Caen, Nancy, Bordeaux, Rennes, and Strasbourg) between January 2013 and June 2019. Active CD at baseline was defined by a Harvey Bradshaw index (HBI) > 4 with at least one objective marker of inflammation: CRP level > 5 mg/ml, faecal calprotectin level > 250 µg/g and endoscopic or radiological signs of disease activity. A group of patients treated with a second anti-TNF agent for active CD was built through the extraction of records from a prospective database from a single centre cohort (Rennes, CNIL No. 1412467). The exclusion criteria were patients exposed to more than one anti-TNF agent, ustekinumab, or vedolizumab prior to inclusion, a time from discontinuation of the first anti-TNF agent to the second-line biological therapy of more than 6 months, and pregnant women.

Failure of the first anti-TNF agent was defined as a primary nonresponse, secondary loss of response, or intolerance. Follow-up was conducted from the date of the initiation of the second-line treatment until the end of the follow-up (last clinical visit), or the date of treatment discontinuation, the need for surgery, or death.

This study was approved by the local IRB: the Hospital Ethics Committee of Rennes (n° 19.78) on July 7, 2019. All methods were carried out in accordance with relevant guidelines, and regulations: Consent form were not required for this type of study and patients were informed (non-opposition letter).

### Data collection

Several data were retrieved from the patients’ charts: gender, age, body mass index (BMI), smoking status, duration of the disease, phenotype classified according to Montreal classification, surgical history, prior immunosuppressant (thiopurines or methotrexate) use, data on the first anti-TNF agent (type of anti-TNF agent (adalimumab or infliximab), date of the first and last injection, reasons for discontinuation, combination with immunosuppressants, optimization prior to discontinuation, last anti-TNF agent through level and antibody level prior to discontinuation), data on the second-line therapy (type of treatment (ustekinumab, vedolizumab or second anti-TNF), date of the first and last injection, combination with corticosteroids and/or immunosuppressants), clinical activity according to HBI, C reactive protein (CRP) levels, haemoglobin levels, faecal calprotectin levels, and endoscopic, and radiological signs of disease activity.

During follow-up, the response to treatment was assessed by clinical activity using the HBI, and objective markers of inflammation, such as CRP levels, faecal calprotectin levels, and endoscopic and radiologic signs of disease activity. At each visit, information on the dose of treatment, combination with immunosuppressants and/or corticosteroids, therapeutic tolerance, and surgical management was collected. When a discontinuation of treatment occurred, the reasons for discontinuation, and duration of treatment were also recorded.

#### Drug regimens

The treatment regimen was administered according to the approved drug. Vedolizumab was started with 300 mg infusions at weeks 0, 2, and 6 as induction therapy, followed by 300 mg infusions every 8 weeks as maintenance therapy. Patients treated with ustekinumab received an induction injection of 6 mg/kg and 90 mg every 8–12 weeks for maintenance therapy. Regarding infliximab, induction therapy was based on infusions of 5 mg/kg at weeks 0, 2, and 6, followed by infusions of 5 mg/kg every 8 weeks as maintenance therapy. Adalimumab was started by an initial 160 mg injection followed by 80 mg, and then 40 mg injection every 2 weeks. Optimization during the maintenance regime was performed at the discretion of the treating physician.

### Outcomes

The primary outcome was the rate of postinduction remission (at weeks 14–24). Remission was defined as clinical remission with HBI < 4 without corticosteroid treatment, and was associated with the absence of an objective marker of inflammation (CRP < 5 mg/L or faecal calprotectin < 250 µg/g or no endoscopic or radiological signs of activity). Patients stopping the treatment during induction were considered as treatment failure.

The secondary outcome was long-term efficacy, as assessed by drug survival. Drug survival was defined by the proportion of patients remaining on the same biological agent, that allowed to assess both tolerance, and efficacy of treatments.

### Statistics

Quantitative variables are described as the means ± standard deviations (SD). Categorical variables are presented as counts, and percentages of the cohort.

For group comparisons, a univariate analysis was performed using the Wilcoxon test for quantitative variables, and a chi-square test or Fisher’s test, as appropriate, for qualitative variables. All significant variables with a *p* value < 0.2 in the univariate analysis were integrated into a binary logistic regression model for the multivariate analysis, and adjusted for baseline differences between groups. When considering the continuous variables for multivariate analysis, cut-off values were determined by performing a receiver operating characteristic (ROC) analysis to reduce the risk of bias related to arbitrarily defined cut-offs, and to identify the optimal cut-off using each outcome as a classification variable. Drug survival, which includes drug efficacy and tolerance, was used to compare the long-term efficacy of biological agents. Variables were censored at the date of biological discontinuation, surgery or last known follow-up. All significant variables with p-values < 0.05 in the log-rank test were retained in the model and integrated into a Cox proportional hazards regression model adjusted for baseline differences between groups. The results are presented as hazard ratios (HRs) with 95% confidence intervals. A *p* value less than 0.05 was considered statistically significant. Statistical analyses were performed using JMP^®^ Pro 13.2.0 software.

## Results

### Baseline characteristics

**Fig. 1:**
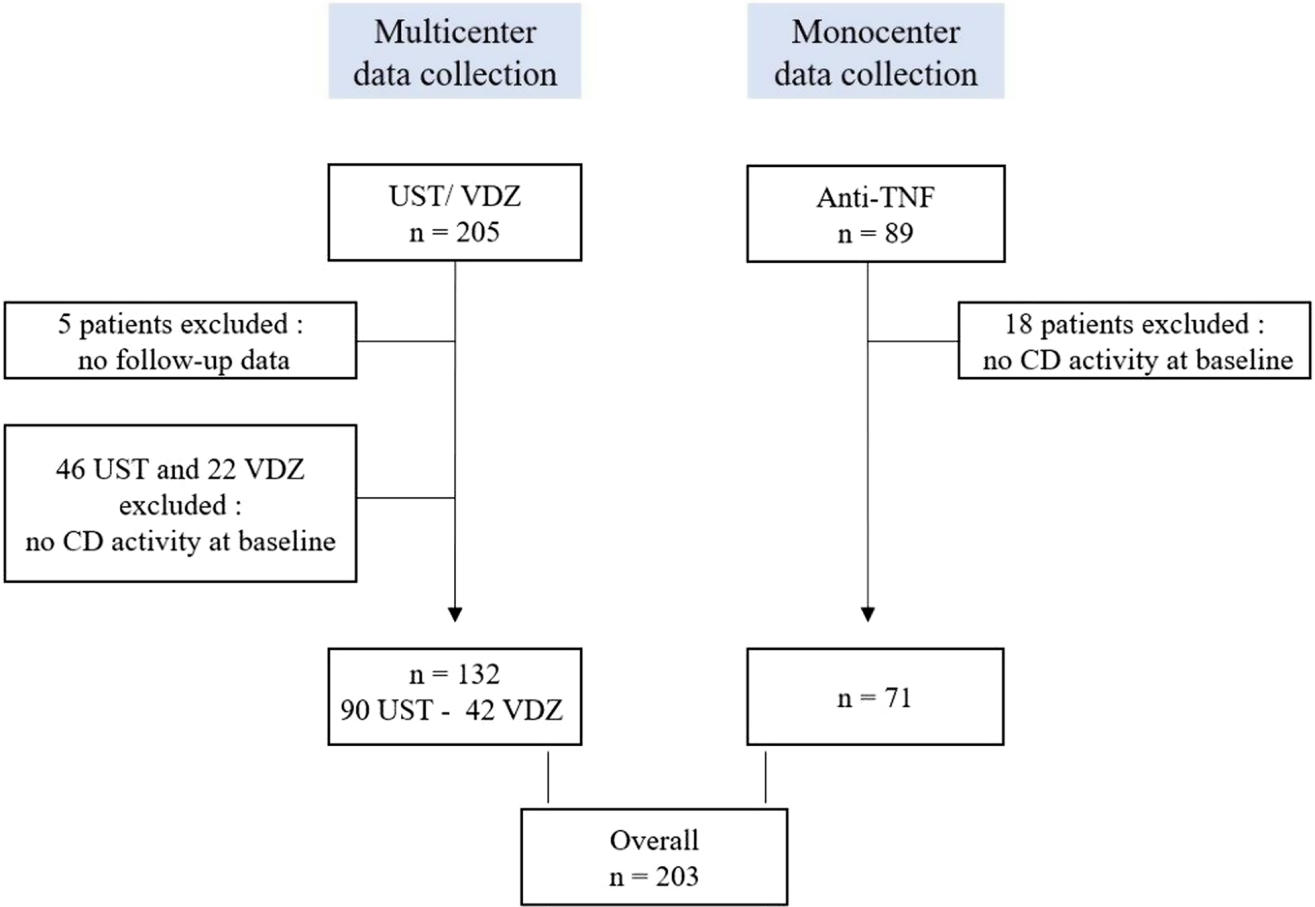
Flow chart of patients with active Crohn's disease (CD) treated with ustekinumab (UST), vedolizumab (VDZ) or anti-TNF after failure of a first anti-TNF agent

Among the 294 patients screened, 86 patients were excluded due to the lack of objective markers of disease activity, and 5 patients were excluded due to a lack of follow-up (Fig. 1). Finally, 203 patients were included, of whom 90 (44%) received ustekinumab, 42 (21%) received vedolizumab and 71 (35%) received a second anti-TNF agent (infliximab or adalimumab).

Baseline characteristics are summarized in Table 1. Eighty-six (44%) patients were male, with a mean age of 38 (± 14) years. The mean disease duration was 10 (± 9) years, 70 (34%) patients had a prior history of a perianal location, and 64 (32%) underwent abdominal surgery. Adalimumab was used as the first-line biologic by 139 (68%) patients, 63 (31%) were treated with infliximab, and 1 (0.5%) was treated with golimumab. The mean duration of anti-TNF treatment was 148 (± 150) weeks, and the treatment was optimized in 124 (61%) patients. Anti-TNF discontinuation was due to a primary nonresponse for 42 (21%) patients and a loss of response or intolerance for 161 (79%) patients. The anti-TNF trough level at discontinuation was available for 130 patients, with a mean of 8.0 (± 7.7) µg/ml.

**Table 1:** Population baseline characteristics

	Overall n = 203	Anti-TNF n = 71 (35%)	Ustekinumab n = 90 (44%)	Vedolizumab n = 42 (21%)	*p*
Male sex, n (%)	86 (44)	31 (44)	37 (44)	18 (43)	0.99
Age (y), mean (± SD)	37.9 (± 14.0)	36.3 (± 14.4)	37.7 (± 13.9)	41.0 (± 13.5)	0.22
BMI (kg/m^2^), mean (± SD)	24.0 (± 5.3)	24.1 (± 6.5)	23.2 (± 3.9)	25.4 (± 4.8)	0.16
Smoking, n (%)	73 (36)	25 (35)	31 (34)	17 (40)	0.79
CD location					0.02
Ileal, n (%)	60 (30)	15 (21)	33 (37)	12 (29)	0.09
Colonic, n (%)	51 (25)	24 (34)	13 (14)	14 (33)	0.0074
Ileocolonic, n (%)	91 (45)	32 (45)	43 (48)	16 (38)	0.58
Upper gastrointestinal disease, n (%)	25 (12)	10 (14)	9 (10)	6 (14)	0.66
CD behaviour					0.004
Non stricturing, non-penetrating, n (%)	121 (60)	55 (77)	44 (49)	22 (52)	0.0007
Strituring, n (%)	48 (24)	10 (14)	28 (31)	10 (24)	0.04
Penetrating, n (%)	34 (17)	6 (8)	18 (20)	10 (24)	0.06
History of perianal disease, n (%)	70 (34)	24 (34)	32 (36)	14 (33)	0.96
History of bowel surgery, n (%)	64 (32)	17 (24)	35 (39)	12 (29)	0.12
History of thiopurines use, n (%)	164 (81)	63 (89)	70 (78)	31 (74)	0.09
*First anti-TNF*					
Combination therapy, n (%)	91 (45)	25 (35)	49 (55)	17 (40)	0.03
Optimization prior discontinuation, n (%)	124 (61)	45 (63)	60 (67)	19 (45)	0.06
Reason for discontinuation					0.27
Primary non-response, n (%)	42 (21)	18 (25)	14 (16)	10 (24)	
Loss of response or intolerance, n (%)	161 (79)	53 (75)	76 (84)	32 (76)	
Anti-TNF concentration, mean (± SD) (n = 130)	8 (7.7)	7.7 (12.5)	8.4 (5.5)	7.4 (4.4)	0.81
< 5 µg/ml, n (%)	44 (34)	21 (62)	17 (25)	6 (21)	0.03
*Second-line therapy*					
Disease activity (mild/moderate/severe), n(%)	87(42)/83(40)/33(16)	35(49)/23(32)/13(18)	39(43)/37(41)/14(15)	13(30)/23(54)/6(14)	0.22
Corticosteroids, n (%)	38 (29)	15 (21)	29 (32)	9 (22)	0.23
Combination therapy, n (%)	73 (36)	50 (70)	17 (19)	6 (15)	< 0.0001
Hb (g/dl), mean (± SD) (n = 144)	13.1 (± 1.5)	13.0 (± 1.5)	13.1 (± 1.5)	13.5 (± 1.3)	0.43
CRP (mg/l), mean (± SD) (n = 180)	18.0 (± 25.5)	18.3 (± 18.1)	21.1 (± 34.6)	11.8 (± 10.8)	0.11

*BMI* body mass index, *CD* Crohn’s disease, *Hb* haemoglobin, *CRP* C-reactive protein

Combination therapy was defined as concomitant use of immunosuppressant therapy (thiopurine or methotrexate). Disease activity was defined according to the Harvey Bradhaw index as mild (HBI 4–8), moderate (HBI 8–12), severe (HBI > 12)

Patients treated with ustekinumab were less likely to have a colonic location (*p* = 0.01), and were more often receiving combination therapy with the first anti-TNF agent than the other groups (*p* = 0.03). B1 behaviour was more frequently observed in patients treated with a second anti-TNF agent (*p* = 0.004). Second-line anti-TNF therapy was more frequently administered in combination with an immunosuppressant (*p* < 0.0001). At inclusion, the mean CRP level was 18.0 (± 25.5) mg/l, with 18.3 (± 18.1) mg/l, 21.1 (± 34.6) mg/l and 11.8 (± 10.8) mg/l recorded for patients treated with a second anti-TNF agent, ustekinumab and vedolizumab, respectively (*p* = 0.11).

### Short-term remission

**Fig. 2:**
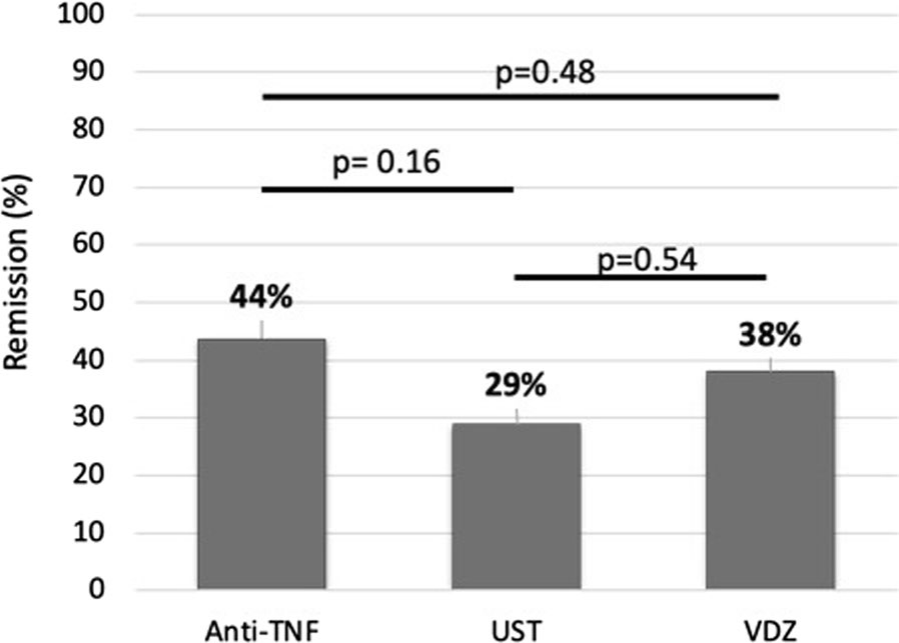
Post-induction remission rate (at week 14–24) according to treatment groups

Short-term efficacy was assessed at the end of the induction phase between weeks 14–24 (mean 19 weeks). The remission rate was not significantly different between patients treated with ustekinumab, vedolizumab, and a second anti-TNF agent (*p* = 0.15) (Fig. 2). The rate of short-term remission within the anti-TNF group according to the primary or secondary failure of the first anti-TNF 35% and 47% (*p* = 0.30), respectively.

Factors associated with short-term remission are depicted in Additional file 1: Table S1. Overall, the use of combination therapy with the first anti-TNF agent was associated with a lower postinduction remission rate (26% vs. 67%, *p* = 0.04, OR = 0.53, 95% CI [0.28–0.99]). This difference was particularly observed for patients treated with vedolizumab (12% vs. 56%, *p* = 0.02, OR: 0.14, 95% CI [0.02–0.79]). For patients treated with a second anti-TNF agent, an ileocolonic location was associated with a higher postinduction remission rate than an ileal location (66% vs. 20%, *p* = 0.01, OR: 7.05, 95% CI [1.51–31.49]), but the opposite result was observed for patients treated with ustekinumab (16% vs. 42%, *p* = 0.01, OR: 0.13, 95% CI [0.03–0.63]).

### Drug maintenance

**Fig. 3:**
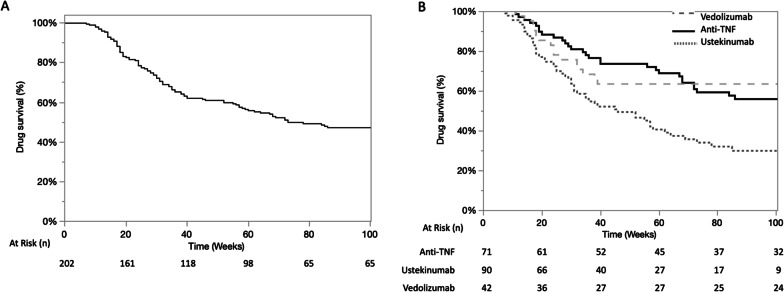
Drug survival for the overall population (**A**) and according to the second-line treatment with anti-TNF, ustekinumab or vedoli zumab (**B**) after failure of a first anti-TNF in CD

Long-term efficacy was assessed by determining the drug survival of the second-line biologic. After a mean follow-up of 118 (± 93) weeks, the cumulative probabilities of second-line biologic survival were 82%, 67%, and 46% at 26, 52, and 104 weeks, respectively. Specifically, the cumulative probabilities at 26, 52, and 104 weeks were 75%, 51%, and 20% for ustekinumab, 86%, 75%, and 57% for vedolizumab, and 87%, 76%, and 58% for the second anti-TNF antibody, respectively (Fig. 3a and b, *p *= 0.0001). After adjustment for the colonic location, behaviour at inclusion, and use of combination therapy, the difference persisted, with decreased drug survival observed for ustekinumab compared to vedolizumab and infliximab (*p* = 0.001).

Factors associated with overall drug survival in different treatment groups are depicted in Additional file 1: Table S2. Regarding the overall population, factors associated with drug survival were tobacco consumption, the absence of optimization of the first anti-TNF treatment prior to discontinuation, and the use of combination therapy at inclusion. The use of combination therapy was particularly associated with drug survival for patients receiving the anti-TNF treatment (*p* = 0.01, HR = 2.37, 95% CI [1.19–4.58]). Looking at the difference between infliximab and adalimumab, the beneficial effect of the combination therapy was only noted for infliximab (*p* = 0.0004) and not for adalimumab (*p* = 0.77).

Discontinuation of the second-line biologic was related to intolerance for 12 (17%) patients treated with a second anti-TNF agent, 4 (4%) patients treated with ustekinumab, and 3 (7%) patients treated with vedolizumab. Serious side effects were reported by 2 patients treated with a second anti-TNF agent (cerebral vasculitis and anal cancer).

### Outcomes stratified according to the anti-TNF trough level prior to second-line therapy

Trough levels prior to the discontinuation of the first anti-TNF were available for 130 (64%) patients. Forty-four (34%) patients had a trough level of the first anti-TNF of less than 5 ng/ml. The mean trough level was 8 (± 7.7) ng/ml for the overall population, 7.7 (± 12.5) ng/ml for patients treated with a second anti-TNF agent (n = 34), and 8.4 (± 5.5) ng/ml, and 7.4 (± 4.4) ng/ml for patients treated with ustekinumab (n = 68), and vedolizumab (n = 28), respectively (*p* = 0.81).

Regarding patients treated with a second anti-TNF agent, 21 (62%) patients presented a level less than 5 ng/ml compared to 25% and 21% of patients treated with ustekinumab, and vedolizumab, respectively (*p* = 0.03).

**Fig. 4:**
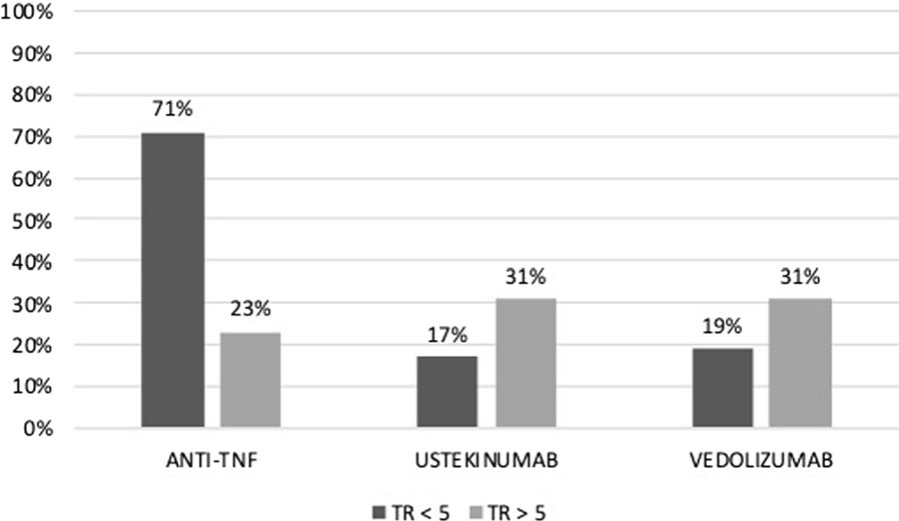
Short-term remission according to the second-line therapy and to the through level of the first anti-TNF agent prior to the discontinuation

The rate of steroid-free remission in the short term for patients treated with a second anti-TNF antibody was 71% in the group with low trough levels (< 5 ng/mL) compared to 18% and 17% in patients treated with ustekinumab, and vedolizumab, respectively (*p* = 0.002). No difference in steroid-free remission in the short term was observed between treatment groups for patients with a trough level greater than 5 ng/mL (*p *= 0.83) (Fig. 4).

**Fig. 5:**
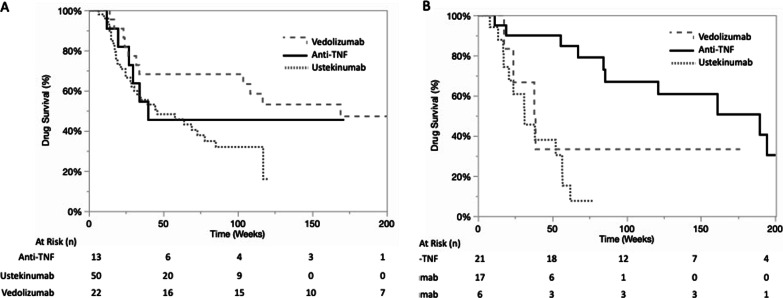
Drug survival according to the second-line therapy and to the through level of the first anti-TNF agent prior to the discontinuation: ≥ 5 µg/ml (**A**) and < 5 µg/ml (**B**)

In the long term, the trough level of the first anti-TNF antibody did not affect the overall drug survival of the second-line biologic (*p* = 0.83). Similar results to the short term were observed after considering treatment groups with a higher drug survival of the second anti-TNF agent who presented a low trough level before switch (*p* = 0.0005), and no difference was observed between groups stratified by the therapeutic trough level (*p* = 0.06) (Fig. 5).

## Discussion

This multicentre retrospective study is the first to directly compare the efficacy of ustekinumab, vedolizumab and a second anti-TNF agent after the failure of a first anti-TNF agent in patients with CD. No significant difference in postinduction remission was observed. Drug survival was shorter for patients receiving the ustekinumab treatment. Few specific factors associated with treatment efficacy were identified to help physicians make for the choice regarding the second-line biologic, except for drug monitoring in patients with low trough levels of the first anti-TNF agent.

Several studies agreed on the absence of differences between these three therapeutic lines in the short term following a drug induction regimen [10, 11, 16, 17]. No difference between vedolizumab, and infliximab was observed in two trials in the short term, including endoscopic remission [18, 19]. Nonetheless, recent studies comparing ustekinumab, and vedolizumab after the failure of anti-TNF agent(s) in patients with CD recommended the use of ustekinumab because of a significantly higher long-term remission rate [10, 11]. The inclusion criteria were different, and the vast majority of patients were previously treated with at least two anti-TNF agents. For both treatments, pivotal trials observed a decreased treatment efficacy when administered to patients with prior exposure to an anti-TNF agent [4, 5]. Specifically, for vedolizumab, a decrease in efficacy was directly correlated with an increase in the number of anti-TNF treatments administered prior to the swap [20]. This result might explain the superiority of ustekinumab in these two retrospective studies, which may help with decisions regarding a third-line biologic. The present study focused on assessing the efficacy of a second-line biologic following the failure of only one anti-TNF agent. The short-term remission rates were 29%, 38%, and 44% for patients treated with ustekinumab, vedolizumab, and a second anti-TNF agent, respectively, consistent with previous reports [10, 21–26].

A few unhelpful factors were associated with treatment efficacy for physician decision making. The use of combination therapy, or optimization during first-line anti-TNF therapy was generally associated with a lower postinduction remission rate and drug survival during second-line therapy (OR = 0.53, 95% CI [0.28–0.99] and OR = 0.52, 95% CI [0.34–0.78], respectively). This finding suggests a lower response rate following the administration of a well-managed first-line biological therapy [26]. The higher drug survival of the combination therapy confirmed the recent study of the addition of an immunosuppressant when switching anti-TNF agents in relapsing patients [27].

Therapeutic drug monitoring of the anti-TNF trough level is known to be helpful for decision making. This study confirmed that the use of an anti-TNF agent remains the best choice in patients with low trough levels rather than swapping the class [28]. The threshold of the trough level of anti-TNF agents was set to 5 µg/ml for both adalimumab and infliximab, according to recent guidelines [29–32]. In contrast, in the case of the optimal through level, a significant difference was not observed between each treatment. In this situation, the use of a second anti-TNF agent allows us to acquire a long-term response for 40% of patients, consistent with a recent study that highlighted the efficacy of therapeutic optimization in patients with a loss of response to infliximab despite a therapeutic through level [33, 34]. Therefore, an optimal anti-TNF though level is not sufficient to guide the therapeutic choice towards a switch to a second anti-TNF agent, or a swap out of the class for ustekinumab or vedolizumab. These results might be partially explained by the presence of undetectable anti-TNF antibodies.

In addition to the retrospective design of the study, patients treated with a second anti-TNF antibody were included in a single centre, which may induce selection bias. This limitation is notably outlined by the baseline difference in patients with more reactive drug monitoring and treated with combination therapy with the second anti-TNF agent, reflecting the centre practice. Baseline characteristics differed between group of treatments, particularly for the disease location and the disease behavior. Non-complicated behavior usually associated with less disabling outcomes was more frequent in the anti-TNF group, which may result in a better response to treatment. Therefore, an analysis was performed after adjusting for confounders to reduce confounding bias. The inclusion criteria were strict to assess the precise question of the efficacy of the second-line biologic. Moreover, this multicentre study was performed in 10 tertiary centres on a large cohort of 203 patients with a long follow-up (mean 118 weeks) during treatment with ustekinumab, and vedolizumab. Objective markers of inflammation were used as selection criteria, and as the primary outcome to avoid the bias associated with a sole subjective assessment of the treating physician. Finally, drug survival was used for the long-term assessment, as it assessed both the efficacy and safety of treatment and results were similar to those observed in a recent nationwide Swedish registers [33].

In conclusion, our study showed no difference in the short term between ustekinumab, vedolizumab, and a second anti-TNF agent, with shorter drug survival observed for ustekinumab, in patients who experienced a loss of response to a first anti-TNF agent. In patients with a low anti-TNF through level prior to the second-line therapy, the use of a second anti-TNF agent displayed superior efficacy. Randomized head-to-head trials comparing different therapeutic strategies are still urgently needed.

## Supplementary Information

**Additional file 1: Table S1. **Factors associated with short-term remission (at weeks 14–24) by univariate and multivariate analysis (adjusted for baseline difference including the disease location, behaviour at inclusion, and use of combination therapy). **Table S2. **Factors associated with drug survival by univariate and multivariate analysis(adjusted for baseline difference including the disease location, behaviour at inclusion, and use of combination therapy).

## Data Availability

The IRB approved to shared anonymized collected data that was noted in the information letter provide to patients. The data underlying this article will be shared on reasonable request to the corresponding author.
