# Correction: Efficacy of ustekinumab, vedolizumab, or a second anti-TNF agent after the failure of a first anti-TNF agent in patients with Crohn’s disease: a multicentre retrospective study

**DOI:** 10.1186/s12876-022-02636-9

**Published:** 2023-02-02

**Authors:** Cassandra Rayer, Maria Nachury, Arnaud Bourreille, Xavier Roblin, Laurent Peyrin-Biroulet, Stephanie Viennot, Mathurin Flamant, David Laharie, Bénédicte Caron, Marie Dewitte, Laurent Siproudhis, Mathurin Fumery, Guillaume Bouguen

**Affiliations:** 1 CHU Rennes, University Rennes, 35000 Rennes, France; 2 CHU Lille, University of Lille, Lille, France; 3 CHU Nantes, Nantes, France; 4 CHU Saint-Etienne, Saint- Étienne, France; 5 Inserm U954 Deparment of Hepato-Gastroenterology, Department of Gastroenterology, Nancy University Hospital, Vandœuvre-Lès-Nancy, France; 6 CHU Caen, Caen, France; 7 Clinique Jules Vernes, Nantes, France; 8 CHU de Bordeaux, Hôpital Haut-Lévêque, Service d’Hépato-Gastroentérologie Et Oncologie Digestive, Université de Bordeaux, 33000 Bordeaux, France; 9 CHU Rennes, University Rennes, INSERM, CIC1414, Institute NUMECAN (Nutrition Metabolism and Cancer), 35000 Rennes, France; 10 Service d’Hépato-Gastroentérologie Et Oncologie Digestive, CHU Amiens Et PeriTox, UMR I0-I, Université de Picardie, Amiens, France; 11 Service Des Maladies de L’Appareil Digestif, 2 Rue Henri Le Guillou, 35033 Rennes Cedex, France

**Correction to: BMC Gastroenterology (2022) 22:498** 10.1186/s12876-022-02583-5

After publication of this article [1], the authors reported that the given and family names of most authors were incorrectly structured. The correct list of authors is: Cassandra Rayer, Maria Nachury, Arnaud Bourreille, Xavier Roblin, Laurent Peyrin-Biroulet, Stephanie Viennot, Mathurin Flamant, David Laharie, Bénédicte Caron, Marie Dewitte, Laurent Siproudhis, Mathurin Fumery and Guillaume Bouguen.

The original article [1] has been corrected.
