# Supplementary material for: Efficacy of ustekinumab, vedolizumab, or a second anti-TNF agent after the failure of a first anti-TNF agent in patients with Crohn’s disease: a multicentre retrospective study
Source: BMC Gastroenterol. 2022 Dec 1;22:498. doi: 10.1186/s12876-022-02583-5 (PMC9717550; doi:10.1186/s12876-022-02583-5)
Supplement: Supplementary file 1 — Additional file 1: Table S1. Factors associated with short-term remission (at weeks 14–24) by univariate and multivariate analysis (adjusted for baseline difference including the disease location, behaviour at inclusion, and use of combination therapy). Table S2. Factors associated with drug survival by univariate and multivariate analysis(adjusted for baseline difference including the disease location, behaviour at inclusion, and use of combination therapy). [file 12876_2022_2583_MOESM1_ESM.docx]

**Supplementary Table 1 –** Factors associated with short-term remission (at weeks 14-24) by univariate and multivariate analysis (adjusted for baseline difference including the disease location, behaviour at inclusion, and use of combination therapy)

|  | **Overall**  **n = 203** | | **Anti-TNF**  **n = 171** | | **Ustekinumab**  **n = 90** | | **Vedolizumab**  **n = 42** | |
| --- | --- | --- | --- | --- | --- | --- | --- | --- |
|  | Univariate  Analysis  p | Multivariate  Analysis  OR [95% CI] - p | Univariate  Analysis  p | Multivariate  Analysis  OR [95% CI] - p | Univariate  Analysis  p | Multivariate  Analysis  OR [95% CI] - p | Univariate  Analysis  p | Multivariate  Analysis  OR [95% CI] - p |
| Gender | 0.76 |  | 0.82 |  | 0.44 |  | 0.17 |  |
| Age (y) | 0.81 |  | 0.17 |  | 0.73 |  | 0.88 |  |
| BMI (kg/m²) | 0.50 |  | 0.61 |  | 0.88 |  | 0.72 |  |
| Smoking | 0.02 | 1.7 [0.90-3.45] – 0.09 | 0.12 | 1.29 [0.42-3.95] – 0.65 | 0.13 | 1.80 [0.52 – 6.23] – 0.33 | 0.32 |  |
| CD location | 0.98 |  | 0.002 |  | 0.03 |  | 0.77 |  |
| Ileal |  |  |  | Reference |  | Reference | 0.76 |  |
| Colonic |  |  |  | 0.6 [0.12 – 2.84] – 0.61 | 0.41 | 1.41 [0.29 – 6.94] – 0.67 | 0.65 |  |
| Ileocolonic |  |  |  | 7.05 [1.57 – 31.49] – 0.01 | 0.01 | 0.13 [0.03-0.63] – 0.01 | 0.47 |  |
| upper GI disease | 0.08 | 0.34 [0.11-1.05] – 0.06 | 0.80 |  | 0.04 | 0.34 [0.06-1.82] – 0.21 | 0.24 |  |
| CD behaviour | 0.18 |  | 0.35 |  | 0.81 |  | 0.36 |  |
| History of perineal disease | 0.11 | 0.69 [0.35-1.35] – 0.28 | 0.45 |  | 0.28 |  | 0.37 |  |
| History of bowel surgery | 0.75 |  | 0.81 |  | 0.60 |  | 0.32 |  |
| History of thiopurines use | 0.26 |  | 0.70 |  | 0.32 |  | 0.39 |  |
| **First anti-TNF** |  |  |  |  |  |  |  |  |
| Combination therapy | 0.009 | 0.53 [0.28-0.99] – 0.04 | 0.97 |  | 0.12 |  | 0.004 | 0.14 [0.02-0.79] – 0.02 |
| Optimization prior discontinuation | 0.09 | 0.84 [0.44-1.59] – 0.59 | 0.50 |  | 0.01 | 0.88 [0.36 – 3.57] – 0.83 | 0.43 |  |
| Reason for discontinuation | 0.26 |  | 0.30 |  | 0.50 |  | 0.54 |  |
| **Second-line therapy** |  |  |  |  |  |  |  |  |
| Corticosteroid | 0.08 | 0.71 [0.34-1.50] – 0.37 | 1.00 |  | 0.09 | 0.34 [0. 06-1.82] – 0.21 | 0.24 |  |
| Combination therapy | 0.94 |  | 0.93 |  | 0.59 |  | 0.04 | All patients are  non-responders |
| Hb (g/dl) | 0.47 |  | 0.82 |  | 0.78 |  |  |  |
| CRP (mg/l) | 0.10 | 1.01 [0.99-1.04] – 0.06 | 0.40 |  | 0.12 |  |  |  |

Abbreviations: BMI, body mass index; CD, Crohn’s disease; GI, Gastrointestinal ; Hb, haemoglobin; CRP, C-reactive protein. Combination therapy was defined as concomitant use of immunosuppressant therapy (thiopurine or methotrexate).

**Supplementary Table 2 –** Factors associated with drug survival by univariate and multivariate analysis(adjusted for baseline difference including the disease location, behaviour at inclusion, and use of combination therapy)

|  | **Overall**  **n = 203** | | **Anti-TNF**  **n = 171** | | **Ustekinumab**  **n = 90** | | **Vedolizumab**  **n = 42** | |
| --- | --- | --- | --- | --- | --- | --- | --- | --- |
|  | Univariate  Analysis  p | Multivariate  Analysis  HR [95% CI] - p | Univariate  Analysis  p | Multivariate  Analysis  HR [95% CI] - p | Univariate  Analysis  p | Multivariate  Analysis  HR [95% CI] - p | Univariate  Analysis  p | Multivariate  Analysis  HR [95% CI] - p |
| Gender | 0.61 |  | 0.60 |  | 0.12 | 0.66 [0.84-2.65] – 0.16 | 0.13 | 1.71 [0.69-4.60] – 0.24 |
| Age (y) | 0.67 |  | 0.44 |  | 0.31 |  | 0.91 |  |
| BMI (kg/m²) | 0.22 |  | 0.32 |  | 1.00 |  | 0.38 |  |
| Smoking | 0.02 | 1.61 [1.05-2.50] – 0.02 | 0.71 |  | 0.006 | 1.93 [0.97 – 4.12] – 0.06 | 0.21 |  |
| CD location | 0.16 |  | 0.72 |  | 0.16 |  | 0.21 |  |
| upper GI disease | 0.13 | 0.49 [0.29-0.87] – 0.01 | 0.41 |  | 0.20 |  | 0.14 | 0.39 [0.14-1.22] – 0.10 |
| CD behaviour | 0.50 |  | 0.21 |  | 0.36 |  | 0.62 |  |
| History of perianal disease | 0.50 |  | 0.87 |  | 0.55 |  | 0.29 |  |
| History of bowel surgery | 0.72 |  | 0.41 |  | 0.38 |  | 0.83 |  |
| History of thiopurines use | 0.60 |  | 0.15 | 0.40 [0.02-2.11] – 0.33 | 0.40 |  | 0.33 |  |
| **First anti-TNF** |  |  |  |  |  |  |  |  |
| Combination therapy | 0.01 | 0.7 [0.48-1.04] – 0.08 | 0.66 |  | 0.39 |  | 0.24 |  |
| Optimization prior discontinuation | 0.001 | 0.52 [0.34-0.78] – 0.002 | 0.29 |  | 0.002 | 0.63 [0.29 – 1.26] – 0.20 | 0.02 | 0.41 [0.14-1.06] – 0.06 |
| Reason for discontinuation | 0.16 |  | 0.13 |  | 0.17 |  | 0.52 |  |
| **Second-line therapy** |  |  |  |  |  |  |  |  |
| Corticosteroid | 0.09 | 0.93 [0.61-1.45] – 0.76 | 0.89 |  | 0.13 | 0.85 [0.47-1.56] – 0.60 | 0.52 |  |
| Combination therapy | 0.01 | 2.26 [1.50-3.47] – 0.0001 | 0.04 | 2.37 [1.19-4.58] – 0.01 | 0.40 |  | 0.39 |  |
| Hb (g/dl) | 0.55 |  | 0.32 |  | 0.65 |  | 0.35 |  |
| CRP (mg/l) | 0.06 | 1.00 [0.99-1.01] – 0.28 | 0.41 |  | 0.43 |  | 0.61 |  |

Abbreviations: BMI, body mass index; CD, Crohn’s disease; GI, gastrointestinal ; Hb, haemoglobin; CRP, C-reactive protein. CD location was defined by the Montreal classification. Combination therapy was defined as concomitant use of immunosuppressant therapy (thiopurine or methotrexate
